# PINK1/Parkin Pathway Activation for Mitochondrial Quality Control – Which Is the Best Molecular Target for Therapy?

**DOI:** 10.3389/fnagi.2022.890823

**Published:** 2022-06-08

**Authors:** Laura F. Silvian

**Affiliations:** Biogen, Cambridge, MA, United States

**Keywords:** Parkinson’s disease, PINK1-Parkin pathway, molecular mechanisms of activation, AMPK, ULK1 (unc-51 like autophagy activating kinase 1), small molecule, cryoEM (cryo-electron microscopy), crystallography

## Abstract

There has been long-term interest in drugging the PINK1-Parkin pathway with therapeutics as a treatment for Parkinson’s disease (PD). Despite significant structural data on Parkin as well as the PINK1 kinase and the multiple conformational changes it undergoes, activation of these targets is non-trivial. This review highlights small molecule screening results that suggests that activation of Parkin biochemically does not necessarily translate to activation of Parkin within cells. There are also issues with activation of PINK1 with kinetin analogs, which do not appear to rescue rodent models of PD. The counter-measure of activating the mitophagy pathway with deubiquitinase (DUB) inhibitors such as USP30 inhibitors is progressing in the clinic for kidney disease and the proof of biology for this target will be tested in these trials. An alternative mechanism of activating Parkin in response to oxidative stress via Parkin phosphorylation by the AMPK-ULK1 pathway may be a simpler way to lower the energy barrier Parkin activation.

## Introduction

In response to oxidative stress, the cell uses signaling pathways to remove damaged mitochondria through autophagy (mitophagy) as an important part of quality control; this process can be dysregulated in diseases such as Parkinson’s disease (PD). Mitochondria are able to metabolize pyruvate from the glycolytic pathway to generate ATP, which is the energy that powers the brain. Mutations in the PINK1 kinase/PARK6 (Valente et al., 2004) or Parkin E3 ligase/PARK2 (Kitada et al., 1998) gene cause early onset autosomal recessive PD due to loss of dopaminergic neurons of the *substantia nigra pars compacta* (SNc). These genetic mechanisms shed light on the nature of the cellular processes that may go awry in idiopathic PD (Yi et al., 2019).

Overwhelming evidence in mammalian cell culture, Drosophila and other models have linked the PINK1/PARKIN to mitophagy, with PINK1 being the sensor of mitochondrial damage. PINK1 and Parkin work together in a feedforward signaling pathway that mediates mitophagy. Under oxidative stress and other damaging insults, PINK1 is stabilized in the outer mitochondrial membrane where it first auto-activates by phosphorylation (Okatsu et al., 2012; Rasool et al., 2018). It then activates mitophagy by phosphorylation of Ubiquitin (pUb) (Kane et al., 2014; Kazlauskaite et al., 2014; Koyano et al., 2014) as well as Parkin on its Ubl domain (pParkin) (Kondapalli et al., 2012; Shiba-Fukushima et al., 2012).

Both PINK1 and Parkin targets are examples of a preclinical therapeutic approach to maintain mitochondrial health in PD patients by activating the mitophagy pathway to improve mitochondrial quality control. An alternative counter-approach toward activating Parkin is to inhibit the mitochondrial deubiquitinase USP30. Knockdown of USP30 rescues the defective mitophagy caused by pathogenic mutations in Parkin (Bingol et al., 2014). Other stress-sensing pathways that involve mitochondrial quality control include the AMPK-ULK1 pathway in which changes to mitochondrial homeostasis can lead to cancer and type 2 diabetes (Cokorinos et al., 2017).

A slate of publications in the last year have increased understanding of the mechanisms of regulation of the PINK1 and Parkin through small molecule therapeutic tools and through crystal and cryoEM structures of PINK1 in different states of activation. Parkin activation has been further elucidated through a high throughput screen to identify small molecule positive allosteric activators that work along with phospho-Ubiquitin to enhance the release of the autoinhibited state (Shlevkov et al., 2022). Furthermore, recent structures of the PINK1 kinase in an activated form from *Tribolium castaneum*, Tc (Rasool et al., 2022) or *Pediculus humanus corporis*, Ph (Gan et al., 2022), have also provided finer details of the step-wise activation mechanism of this kinase.

## Parkin as a Therapeutic Target

**FIGURE 1:**
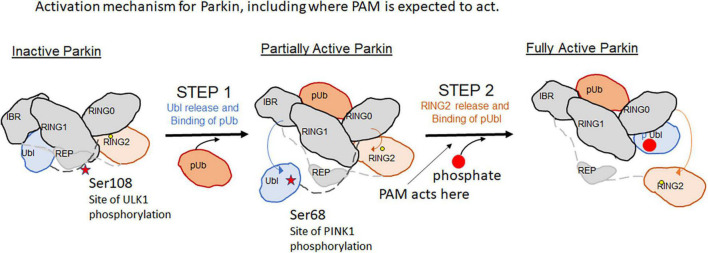
Activation mechanism for Parkin, including where PAM is expected to act.

Parkin is a cytosolic Ring-between-Ring E3 ligase that uses a hybrid mechanism of ubiquitin transfer. It does not direct Ubiquitin transfer onto substrate lysines directly, instead the reaction must go through a catalytic cysteine intermediate before transfer to substrate lysines. Parkin’s activity is highly regulated– phosphorylation of the Ubiquitin-like (Ubl) domain by PINK1 as well as binding of phospho-Ubiquitin is necessary to fully release its autoinhibited conformation (Figure 1). Once activated, the RING2 domain of Parkin containing the catalytic active site cysteine is released and is available for catalytic transfer of Ubiquitin (Tang et al., 2017).

In early studies, researchers seeking small molecules that “activate” Parkin used assays that monitored cellular mitophagy in response to oxidative stress (Garofalo et al., 2017; Johnston and Garofalo, 2017; Springer et al., 2018; Shiba-Fukushima et al., 2020). However, these assays were prone to identifying molecules that actually damage mitochondria, not true Parkin activators, though both types of compounds would have the same readout.

In a recent study, a high throughput *in vitro* biochemical assay was assembled to identify small molecule positive allosteric modulators (PAMs) that worked in conjunction with phospho-Ubiquitin to enhance auto-Ubiquitination of wildtype Parkin (Shlevkov et al., 2022). Several series of compounds were confirmed, and the best compound series (tetrahydropyrazolo pyrizine scaffold) was optimized for potency. The best active compound described (BIO-2007817) achieved the same level of activation as the W403A Parkin positive control, and achieved an Ec_50_ in the ∼100 nM range. It is likely interacting specifically to Parkin as the enantiomer of this compound was inactive in the biochemical assays, suggesting that there is a single binding site. The compounds identified do not accelerate Parkin ubiquitination in the absence of pUb, but do also enhance pParkin autoubiquitination, though to a lesser extent. Using a Parkin-Ub-Vinylsulfone assay to identify the minimal components that the compounds act on, the tools appear to be directly activating Parkin, and not the E1 or E2 present in the original biochemical assay.

However, the compounds did not accelerate mitophagy or accelerate Parkin translocation to mitochondria. Phospho-poly-Ub expression on mitochondrial proteins promotes the translocation of Parkin to mitochondria and serves as an important cellular assay of activation (Shiba-Fukushima et al., 2014). The active compounds did not accelerate Parkin translocation, in contrast to the W403A mutant Parkin, which accelerates Parkin translocation to mitochondria and served as a positive control. Similarly, the compounds failed to enhance mitophagy. The proposed explanation advanced is that these PAMs act on Parkin at a step after the rate-limiting step within the cell, thus their effect appears to be negligible on Parkin translocation or the downstream mitophagy readout.

## PINK1 as a Therapeutic Target

PINK1 (PTEN-induced putative kinase 1) is a highly conserved kinase that is activated by mitochondrial membrane potential depolarization to phosphorylate Ubiquitin and Parkin; its activity on these two substrates triggers the feed-forward loop to mitophagy (Kondapalli et al., 2012). PINK1 has 3 insertions in the N-terminal domain (inserts 1, 2, and 3) that distinguish it from other kinases.

Two recent studies of activated dimeric PINK1 by crystallography (Rasool et al., 2022) and Cryo electron microscopy (Gan et al., 2022) have demonstrated a putative mechanism of kinase activation caused by oxidation and phosphorylation. PINK1 accumulates on the outer mitochondrial membrane in response to mitochondrial depolarization and its interaction with the translocase of the outer membrane (TOM complex) (Tokarew et al., 2021) leads to autophosphorylation of Thr257 as well as Ser228 of the C-loop and Ser402 of the activation loop. The structures demonstrate that dimeric interface of PINK1 requires insert2 and that phosphorylation of the C-loop as well as ordering of insert 3 is necessary for conformational changes in PINK1 required to bind Ubiquitin.

The order of activation is detailed as follows: First, cysteine oxidation in the P-loop of the active site (Cys166 in hPINK1) acts as a redox switch and is responsible for stabilizing the PINK1 dimer. Second, Ser228 within the C-loop of one monomer is phosphorylated which enables a conformational change of the alpha-C helix and structures insert3 to prime it to bind Ubiquitin. Then the second monomer of the dimer is phosphorylated and also primes it to bind Ubiquitin. Third, the PINK1 dimer dissociates into monomers as regulated by reactive oxygen species and Ub can now access and bind to the unique conformation of the autophosphorylated PINK1 N-terminal domain. The Ser65 of the substrate (Ubiquitin or the equivalent residue in the Parkin Ubl) is placed in the active site of the kinase, where it is phosphorylated.

**FIGURE 2:**
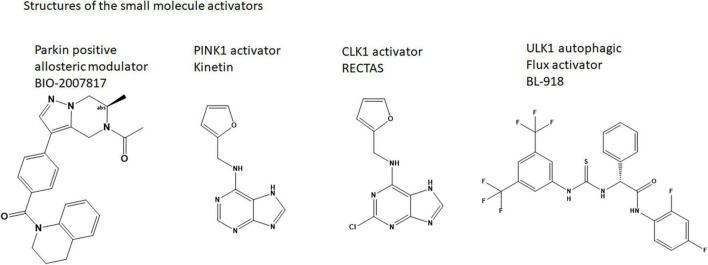
Structures of the small molecule activators.

A neosubstrate kinetin (N6-Furfuryladenine; a plant hormone related to Adenosine; Figure 2), has been shown to increase the rate of autoactivation of PINK1, increase Parkin’s recruitment to depolarized mitochondria, and block mitochondrial motility in axons (Hertz et al., 2013; Osgerby et al., 2017). Kinetin-like analogs have been designed to improve the activation of PINK1—as exemplified in WO2021 1168446 A1. Using the ProTide prodrug technology, these authors showed that a nucleoside monophosphate is the active species for activation (Osgerby et al., 2017). It is tempting to hypothesize that these compounds stabilize the PINK1 dimeric species by binding to its active site and enabling the conformational changes to prime the kinase for Ub binding. A similar compound, RECTAS, a Chloride analog of Kinetin (Figure 2), is known as a splice modulator compound that works by activating CLK1, resulting in phosphorylation of the SR (arginine-serine-rich) protein of splicing factors, which leads to splice site inclusion of the IKBKAP-FD exon 20 (Ajiro et al., 2021). While it is not clear exactly how RECTAS activates CLK1, CLK1 also has a unique, flexible N-terminal domain that enables oligomerization and specific binding to RS-domain containing substrates.

Despite its promise as a small molecule activator of PINK1, long-term studies of oral kinetin has been shown to not protect against synuclein-induced neurodegeneration in rodent models of PD (Orr et al., 2017).

### USP30 as a Therapeutic Target

The counter-strategy to activating Parkin and enhancing the mitophagy pathway is to inhibit enzymes that de-ubiquitinate mitochondrial protein. Deubiquitinating enzymes (DUBs) such as USP30 (Bingol et al., 2014), USP8 (Mizuno et al., 2005 #143; Durcan et al., 2014; #141) USP15 (Cornelissen et al., 2014, 2018), and Ataxin-3 (Durcan and Fon, 2011; Durcan et al., 2012) have been identified as ones that deubiquitinate mitochondrial proteins as summarized recently (Chakraborty and Ziviani, 2020). One of the challenges in this as a therapeutic approach is the difficulty of blocking many distinct DUBs. The pleotropic nature of this deubiquitination phenomenon means that it may not be possible to target one DUB without others substituting in its place.

USP30 and Parkin have common targets such as TOM20 and MIRO1 and USP30 has a negative feedback effect on Parkin. Several UPS30 inhibitors have been advanced and are recently reviewed (Wang et al., 2022). The most advanced compounds are substituted cyanopyrrolidines which has been reported as entering clinical studies by Mission Therapeutics (US20180086708A1). Their first DUB program (MTX652) is being tested in kidney disease starting in April 2022. If successful in proof of biology, a brain-penetrant DUB may be required for testing in Parkinson’s disease. FORMA therapeutics similarly has generated patents for cyanopyrrolidine inhibitors of USP30 (US20200317658). Genentech also has described Usp30 inhibitors (US2014041111A1). Mitobridge, acquired by Astellas, has also generated USP30 inhibitors such as M-094, a benzenesulonamide which appears to be very specific for USP30 (Kluge et al., 2018). The efficacy and safety of DUB inhibitors to act as proof-of-concept therapy to improve mitochondrial health remains to be tested in the clinic.

## AMPK-ULK1 as a Therapeutic Target

Interestingly, Parkin also serves to connect downstream mitophagy to the AMPK-ULK1 pathway, which senses cellular stress. AMPK activates ULK1 through physical interaction and phosphorylation of multiple sites, which results in translocation of ULK1 to the mitochondria (Laker et al., 2017). Recent studies demonstrate that ULK1 (UNC-51-like kinase 1) phosphorylates the ACT domain within Parkin, resulting in enhanced mitophagy (Hung et al., 2021).

In fact, mutation of a 3 residue cluster Ser108/Ser109/Ser110 within the Parkin ACT domain to Alanine or Threonine leads to delays in the rate of mitophagy. In particular, Ser 108 is the substrate for phosphorylation by ULK1. The ACT domain was previously shown to be disordered in the autoinhibited Parkin structure; but in the active pParkin-pUb structure, this region is thought to enable Ring2 release by mimicking interactions of Ring2 on the UPD and shielding the UPD once the Ring2 domain is released. Looking at greater detail at the phospho-Parkin/pUb complex structure (Gladkova et al., 2018)(PDBID:6GLC), while the Ser108 residue was poorly ordered, it docks near some highly basic residues like R256, W183. If phosphorylation of Ser108 activates Parkin, it is possible that a unique pocket for the phosphorylated Ser108 could help fully stabilize the Ring2-released, activated state of Parkin. The Hung et al. (2021) study suggests that even small amounts of mitochondrial stress can cause quick phosphorylation of this site within 5 min (Hung et al., 2021) unlike activation of PINK1 which occurs 30–60 min after mitochondrial damage. Thus the phosphorylation of the ACT domain by ULK1 is an early “alert signal” of mitochondrial damage.

This connection suggests that activation of AMPK, which senses cellular stress, could also be a potential therapeutic approach for neurological disease such as PD. An oral AMPK activator, Metformin, is approved for type 2 diabetes and reduces the risk of cancer. Studies have shown that metformin can also penetrate the blood-brain barrier and protect neurons. Repurposing of anti-diabetic agents for the treatment of cognitive impairment and mood disorders has been proposed (Cha et al., 2016).

Potentially even more specifically as a Parkinson’s disease therapy, activation of ULK1 by a small molecule may sensitize Parkin for activation and enable mitophagy. A small molecule (33i, BL-918) (Figure 2) has been identified. The compound enabled autophagic flux in SH-SY5Y cells and reversed MPP + induced cell death (Ouyang et al., 2018). It is possible that this compound is also indirectly activating Parkin.

## Discussion

In summary, the cell uses multiple regulation mechanisms to ensure that damaged mitochondria are cleared. By generating tool compounds and testing phosphorylation sites in PINK1 and Parkin, the field is getting closer to a molecular understanding of how dysregulation of this pathway could lead to dopaminergic neuron loss in PD or poor mitochondrial clearance in diabetes. But therapeutic agents that can reverse the damage by activating the pathway are in short supply. There are disconnects between *in vitro* biochemical and cell-based biochemical outcomes for Parkin activators, and disconnects between cell-based and mouse model outcomes for PINK1 activators. While it is generally accepted that activation of a target downstream in the cascade provides the better specificity than activating targets upstream, possibly the best therapeutic approach requires a pivot from activating Parkin to activating ULK1 or AMPK upstream.

## Author Contributions

The author confirms being the sole contributor of this work and has approved it for publication.

## Conflict of Interest

LS is employed by company Biogen.

## Publisher’s Note

All claims expressed in this article are solely those of the authors and do not necessarily represent those of their affiliated organizations, or those of the publisher, the editors and the reviewers. Any product that may be evaluated in this article, or claim that may be made by its manufacturer, is not guaranteed or endorsed by the publisher.
